# Association of patterns of multimorbidity with length of stay: A multinational observational study

**DOI:** 10.1097/MD.0000000000021650

**Published:** 2020-08-21

**Authors:** Carole E. Aubert, Jeffrey L. Schnipper, Niklaus Fankhauser, Pedro Marques-Vidal, Jérôme Stirnemann, Andrew D. Auerbach, Eyal Zimlichman, Sunil Kripalani, Eduard E. Vasilevskis, Edmondo Robinson, Joshua Metlay, Grant S. Fletcher, Andreas Limacher, Jacques Donzé

**Affiliations:** a Department of General Internal Medicine, Inselspital, Bern University Hospital, University of Bern, Switzerland; b Institute of Primary Health Care (BIHAM), University of Bern, Bern, Switzerland; c Center for Clinical Management Research, Veterans Affairs Ann Arbor Healthcare System, Ann Arbor, MI; Institute for Healthcare Policy and Innovation, University of Michigan, Ann Arbor, MI, USA; d BWH Hospitalist Service, Division of General Medicine, Brigham and Women's Hospital, Boston, MA; e Harvard Medical School, Boston, MA; f CTU Bern, and Institute of Social and Preventive Medicine, University of Bern, Bern, Switzerland; g Division of Internal Medicine, Lausanne University Hospital, Lausanne, Switzerland; h Department of Internal Medicine, Geneva University Hospital, Geneva, Switzerland; i Division of Hospital Medicine, University of California, San Francisco, CA; j Sheba Medical Centre, Tel Hashomer, Israel; k Section of Hospital Medicine, Division of General Internal Medicine and Public Health Vanderbilt University, Nashville, TN; l Center for Clinical Quality and Implementation Research, Vanderbilt University, Nashville, TN; m VA Tennessee Valley, Geriatric Research, Education and Clinical Center, Nashville, TN; n Christiana Care Health System, Wilmington, DE; o Division of General Internal Medicine, Massachusetts General Hospital, Boston, MA; p Department of Medicine, Harborview Medical Center, University of Washington, Seattle, WA.; q Division of General Internal Medicine and Primary Care, Brigham and Women's Hospital, Boston, MA; r Department of Internal Medicine, Neuchâtel Hospital Network, Neuchâtel, Switzerland.

**Keywords:** chronic diseases, combinations, health care utilization, length of stay, multimorbidity

## Abstract

The aim of this study was to identify the combinations of chronic comorbidities associated with length of stay (LOS) among multimorbid medical inpatients.

Multinational retrospective cohort of 126,828 medical inpatients with multimorbidity, defined as ≥2 chronic diseases (data collection: 2010–2011). We categorized the chronic diseases into comorbidities using the Clinical Classification Software. We described the 20 combinations of comorbidities with the strongest association with prolonged LOS, defined as longer than or equal to country-specific LOS, and reported the difference in median LOS for those combinations. We also assessed the association between the number of diseases or body systems involved and prolonged LOS.

The strongest association with prolonged LOS (odds ratio [OR] 7.25, 95% confidence interval [CI] 6.64–7.91, *P* < 0.001) and the highest difference in median LOS (13 days, 95% CI 12.8–13.2, *P* < 0.001) were found for the combination of diseases of white blood cells and hematological malignancy. Other comorbidities found in the 20 top combinations had ORs between 2.37 and 3.65 (all with *P* < 0.001) and a difference in median LOS of 2 to 5 days (all with *P* < 0.001), and included mostly neurological disorders and chronic ulcer of skin. Prolonged LOS was associated with the number of chronic diseases and particularly with the number of body systems involved (≥7 body systems: OR 21.50, 95% CI 19.94–23.18, *P* < 0.001).

LOS was strongly associated with specific combinations of comorbidities and particularly with the number of body systems involved. Describing patterns of multimorbidity associated with LOS may help hospitals anticipate resource utilization and judiciously allocate services to shorten LOS.

## Abstract 2

Supplemental Digital Content is available in the text

## 1 Introduction

The length of hospital stay (LOS) is a commonly used measure of health care resource utilization.^[1]^ Although it is directly associated with health care costs in countries with a charging system based on the number of hospital days, it has gained further interest from health care providers in countries with a charging system based on diagnosis-related groups.^[2]^ Previous studies showed that the LOS could be efficiently reduced through different interventions, such as twice-daily consultant ward round, interdisciplinary geriatric care, functional maintenance program, or nurse-led interventions.^[3,4]^ It is therefore important to identify factors associated with a longer LOS so that such services could potentially be targeted.

Multimorbidity, assessed simply as a number of conditions or with the Charlson Comorbidity Index, has been associated with a longer LOS.^[5–8]^ Furthermore, some data suggest that multimorbidity is associated with long-term dependency, which in turn can increase the risk of hospitalization and delay discharge, thus extending the LOS.^[9]^ However, which combinations of chronic comorbidities are associated with a longer LOS has not been well studied. Little is known as well about some potentially more complex effects of multimorbidity on the LOS, such as interactions between comorbidities that may have multiplicative effects on the LOS.

The primary aim of our study was to identify combinations of chronic comorbidities associated with LOS in multimorbid medical inpatients, to quantify this association, and to assess potential multiplicative effects of these comorbidities on the LOS. The secondary aim was to quantify the association between the LOS and the number of chronic diseases and body systems involved.

## 2 Methods

### 2.1 Study design, setting and population

We used a retrospective cohort of medical inpatients aged ≥18 years, with multimorbidity (defined as ≥2 chronic diseases)^[10,11]^ and discharged home or to a nursing home from 11 large hospitals across three countries (7 hospitals in the United States, 3 hospitals in Switzerland, and 1 hospital in Israel) during calendar years 2010 to 2011 (detailed list of hospitals in Appendix). The cohort was initially built to study readmission.^[12]^ All data, including *International Classification of Diseases* (*ICD*) diagnosis codes available at discharge, were extracted from electronic medical records. The ethical committee of each participating site approved the data collection. Because this study falls under a further use of existing fully anonymized data, the study was exempt from further Institutional Review Board review. This study is in accordance with the STrengthening the Reporting of OBservational studies in Epidemiology (STROBE) statement.^[13]^ The dataset is not publically available, but can be requested from the corresponding author on reasonable request (caroleelodie.aubert@insel.ch).

### 2.2 Classification of diseases

We included only chronic diseases according to the Chronic Condition Indicator of the Healthcare Cost and Utilization Project (HCUP), a Federal-State-Industry partnership sponsored by the Agency for Healthcare Research and Quality.^[14]^ This tool defines a chronic disease as a condition lasting at least 12 months and placing limitations on self-care, independent living and social interactions and/or resulting in the need for ongoing intervention with medical products, services, and special equipment. We grouped all *ICD* codes classified as chronic diseases into a clinically meaningful and more reasonable number of comorbidities using the Clinical Classification Software of the HCUP, and into 18 body system categories using the Chronic Condition Indicator (details in Appendix).^[14,15]^ As previous authors, we excluded *ICD* codes for screening strategies, symptoms, risk factors, and complications.^[16,17]^ For clinical relevance, we further collapsed some comorbidities together (details in Appendix).

### 2.3 Outcome

The outcome was the LOS, defined as the number of days from hospital admission to hospital discharge. We used it as a binary or as a continuous variable, depending on the analyses. Because the LOS varied by country (e.g., longer in Switzerland than in the United States), we defined a prolonged LOS as a LOS longer than or equal to the country-specific upper (75%) quartile.^[18,19]^

### 2.4 Statistical analyses

We present baseline characteristics as median with interquartile range (IQR) or frequencies with percentage, as appropriate. For the primary aim, we first used a mixed-effects logistic univariable regression with a random intercept for center to identify the 20 combinations of comorbidities with the highest odds ratio (OR) for a prolonged LOS, comparing patients with, to those without the combination. Then, for these 20 combinations, we assessed the difference in median LOS using quantile regression controlled for center and calculated the attributable LOS, obtained by multiplying the difference in median LOS by the number of patients having the combination of comorbidities. Finally, we analyzed the interactions among the comorbidities of each of these 20 combinations on the odds of a prolonged LOS in mixed-effects logistic regression and displayed the results as a nonsignificant interaction, a more than multiplicative effect, or a less than multiplicative effect. A more than multiplicative effect means that the OR of the combination is higher than the ORs of the single comorbidities multiplied together.

For the secondary aim, we used a mixed-effects logistic univariable regression with a random intercept for center to quantify the association between a prolonged LOS and the number of chronic diseases, and the number of body systems involved, and presented the results in forest plots.

We performed all analyses with STATA 15.1 (StataCorp LP, College Station, TX) or R version 3.4.4 (R Project for Statistical Computing).

## 3 Results

**Figure 1:**
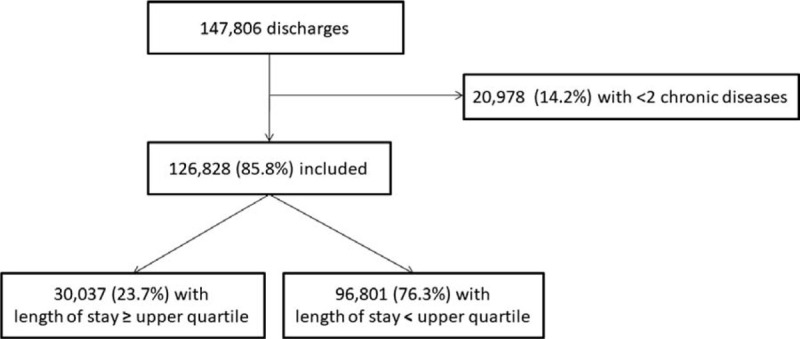
Study flow-chart.

Among the 147,806 discharged patients available in the initial cohort, 126,828 (86%) had multimorbidity and were included for analysis (Fig. 1). Table 1 presents the baseline characteristics of the patients in the whole population and according to prolonged LOS. Median age was 64 years (IQR 52–76), median number of chronic diseases was 5 (IQR 3–8), and median LOS was 5 days (IQR 3–8). The most frequent comorbidities were chronic heart disease and chronic kidney disease, with a prevalence of 48% and 18%, respectively.

**Table 1:**
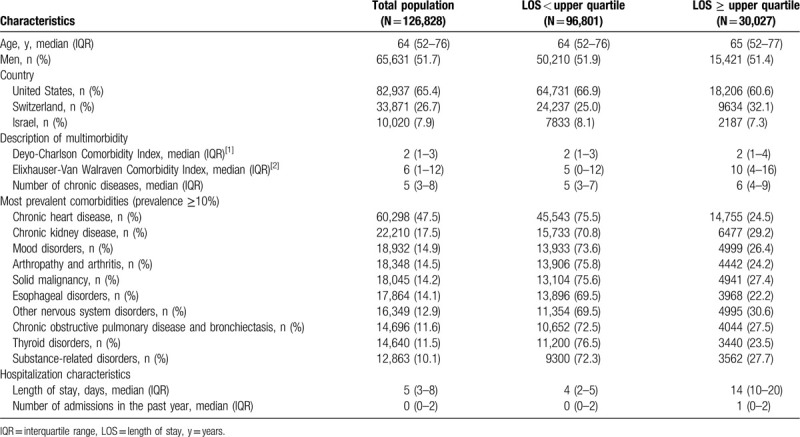
Baseline characteristics.

### 3.1 Combinations of comorbidities and LOS

Diseases of white blood cells combined with hematological malignancy showed the highest OR for a prolonged LOS (7.25, 95% confidence interval [CI] 6.64–7.91), the highest difference in median LOS (13 days, 95% CI 12.8–13.2), and the highest attributable LOS (29,744 days; Table 2). The 19 following combinations increased the odds of a prolonged LOS by 137% to 265%, had a difference in median LOS of 2 to 5 days, and an attributable LOS of 1280 days for chronic ulcer of skin with peripheral and visceral atherosclerosis, to 10,248 days for chronic ulcer of skin with chronic heart disease. These 19 combinations included mostly neurological diseases and chronic ulcer of the skin. The comorbidities of 5 of the combinations had a more than multiplicative effect on the odds of a prolonged LOS, whereas those of 6 combinations had a less than multiplicative effect.

**Table 2:**
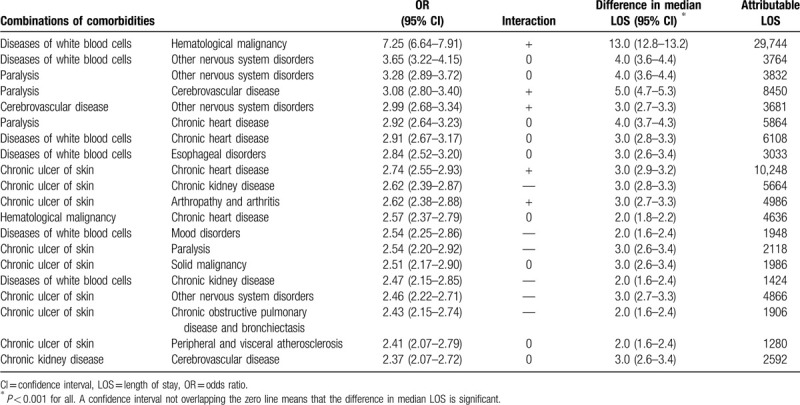
Combinations of comorbidities and length of stay.

### 3.2 Number of chronic diseases, number of body systems involved, and LOS

**Figure 2:**
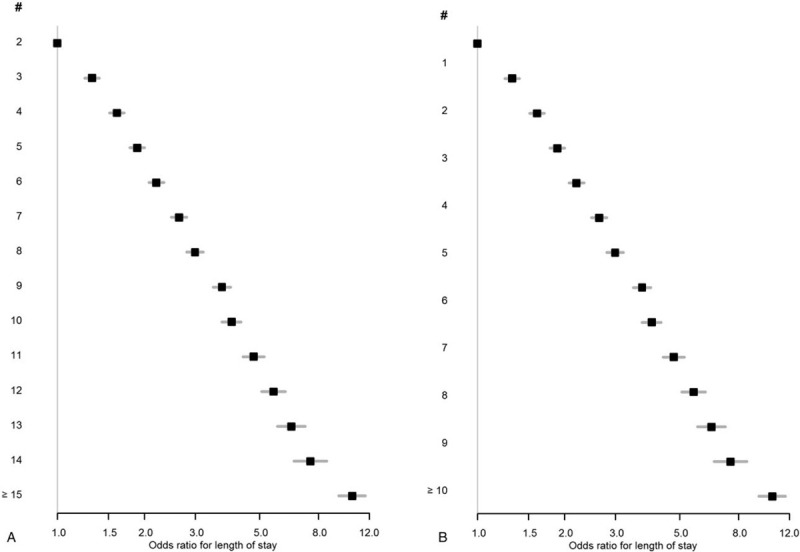
Association between prolonged length of stay and (A) number of chronic diseases and (B) number of body systems involved. #, number of chronic diseases (A)/body systems (B). Odds ratio (box) with 95% confidence interval (lines) for prolonged length of stay, defined as a length of stay longer than or equal to country-specific upper (75%) quartile.

Most patients had 3 or 4 chronic diseases (prevalence 14% for each) and 3 body systems involved (prevalence 21%). The OR for a prolonged LOS exponentially increased with the number of chronic diseases and particularly with the number of body systems involved; the OR was 5.33 (95% CI 5.03–5.64) for patients with ≥10 chronic diseases, and 21.50 (95% CI 519.94–23.18) for those with ≥7 body systems involved (Fig. 2, details in Appendix).

## 4 Discussion

In this large multinational cohort of medical multimorbid inpatients, we identified combinations of chronic comorbidities associated with a prolonged LOS, quantified this association, and assessed multiplicative effects of the comorbidities on the LOS. Diseases of white blood cells with hematological malignancy increased the odds of a prolonged LOS by 625% and had the largest difference in median LOS (13 days). Other combinations of comorbidities associated with prolonged LOS included mostly neurological diseases and chronic ulcer of skin, and increased the odds by 137% to 265%. One-fourth of the combinations of comorbidities increased the odds of a prolonged LOS more than expected, as revealed by the interaction analysis. The association between LOS and the number of body systems was particularly strong, reaching an OR of >20 for patients with ≥7 body systems involved. This study identified chronic comorbidities with the highest impact on LOS and pointed out the importance of the number of chronic diseases and of body systems involved on the LOS.

### 4.1 Combined comorbidities and LOS

With an odds of a prolonged LOS increased by 625% and a difference in median LOS of 13 days for the combination of diseases of white blood cells with hematological malignancy, the association was far stronger than for other combinations; the second top combination showed an association less than half as strong, with an OR of 3.65 and a difference in median LOS of 4 days only. This strong association and particularly large difference in median LOS could be mostly explained by the neutropenia related to the hematological malignancy itself and/or occurring as an expected consequence of the aplasia period resulting from chemotherapy.^[20]^ Previous authors indeed found a median LOS of 16 days in patients with a hematological malignancy and febrile neutropenia.^[21]^ The LOS may unfortunately be difficult to shorten, first because these patients most often must stay in an isolated hospital room until bone-marrow recovery occurs, and second because of the almost unavoidable complications of aplasia, such as severe mucositis, that may prevent enteral nutrition, and infections that most often occur despite prophylactic drugs and strong hygiene practices. Although one may argue that hematological malignancy and diseases of white blood cells are part of a single pathology, they are classified in different CCS and were therefore considered as different comorbidities.

Neurological diseases combined together or with other comorbidities were frequently identified in the next combinations with the strongest association with a prolonged LOS. There may be different explanations for this finding, partly depending on the specific neurological diseases. First, for cerebrovascular diseases, it may depend on the kind of care during hospitalization, as inhospital interventions such as stroke units or care by neurohospitalists could shorten the LOS in patients with stroke.^[22,23]^ Second, neurological diseases often result in disability, increasing patients’ dependency and consecutive need for additional supportive home care or discharge to a nursing home with specialized care for paralysis or tracheotomy for example. This might require some time to arrange due to limited resources, which is a possibly modifiable factor. Third, we excluded patients discharged to a rehabilitation facility and may thus hypothesize that those patients with a neurological disease and prolonged LOS included in our study may have been more severely affected or too ill to qualify for rehabilitation care.

Diseases of white blood cells and chronic ulcer of the skin were also very often found in the top combinations. Both comorbidities are associated with an increased risk of infection and may thus lead to complications prolonging LOS. Furthermore, chronic ulcer of skin may require daily care that is not always easy to organize outside of the hospital. Altogether, both comorbidities may be seen as markers of frailty and of multimorbidity severity.

The top 3 combinations with comorbidities increasing the odds of a prolonged LOS more than expected (more than multiplicative effect) included 2 combinations of neurological diseases, as well as the combination of diseases of white blood cells with hematological malignancy. Although we may have only little influence on the LOS related to the latest, as discussed above, particular attention should be focused on patients with combinations of neurological diseases, as efficient interventions may shorten the LOS in these patients.^[3,22,23]^ The other 2 combinations with comorbidities with a more than multiplicative effect both included chronic ulcer of the skin, underlying the importance of adequate care to prevent this potentially avoidable complication. It is worth noting that almost one-third of the comorbidities found in the 20 top combinations had a less than multiplicative effect on the odds for a long LOS, despite the exponential relationship that we found with the number of diseases. This suggests that the number of diseases may thus itself not be sufficient to assess the real burden of multimorbidity.

### 4.2 Number of chronic diseases, number of body systems involved, and LOS

Previous authors have described a longer LOS associated with the number of diseases, with the Deyo-Charlson Comorbidity Index or with multimorbidity assessed as present or absent, but none had quantified this relationship with the number of chronic diseases or body systems involved.^[5,6,8,24]^ However, one study found no significant difference in LOS between patients with versus those without multimorbidity, but this study was small (N = 413), considered only 18 diseases in the assessment of multimorbidity and included only surgical patients aged 65 years or older, limiting the generalizability and comparability of the results with our analyses.^[25]^ We found a particularly strong association between the LOS and the number of body systems involved, with an OR reaching >20 for patients with ≥7 body systems involved, suggesting that the number of body systems involved, in addition to or instead of the number of diseases, may simplify the assessment of the association between multimorbidity and adverse health outcomes.

### 4.3 Implications and explanations

The identification of combinations of comorbidities associated with a prolonged LOS is important to identify patients most likely to have a prolonged LOS. This may help hospitals to focus on specifically high-risk patients to decrease the burden of multimorbidity and hospitalizations. Furthermore, the assessment of combinations of comorbidities, and not only of multimorbidity as a simple number of diseases, may help to focus on multimorbid patients with potentially modifiable LOS, since not everything can be influenced, as discussed for example for complications of aplasia in patients with hematological diseases, and to develop focused interventions according to specific diseases that are most likely responsible for prolonging the LOS. Finally, the quantification of the associations performed in this study may further help to set priorities when aiming to shorten the LOS. Altogether, this descriptive and quantitative detailed assessment of multimorbidity may help to identify patients on which we should primarily focus preventive interventions to shorten the LOS, with the final goal to reduce the burden of multimorbidity and health care costs without decreasing quality of care.

### 4.4 Strengths and limitations

Our study presents some limitations. First, although we could assess a broad number of diseases using ICD-codes, diagnoses are subject to coding quality, so that we cannot exclude some underreporting.^[26,27]^ Second, our study was not designed to assess causal relationships, so that remaining confounders are possible and we can only describe associations. Finally, we studied medical patients only, so that we cannot generalize our findings to surgical patients, whose LOS may be further affected by other reasons such as surgical complications.

This study has a number of strengths also. First, we used a large multinational cohort, increasing results’ generalizability. Second, we used standardized tools to define and classify the diseases.^[14,15]^ Third, unlike most previous studies limiting the number of diseases to a specific and often subjective selection, we considered all *ICD*-coded diagnoses. Finally, we assessed multimorbidity using different innovative measures such as combinations of comorbidities, number of chronic diseases and number of body systems involved, and further studied multiplicative effects.

### 4.5 Conclusions, recommendations and future directions

In this large multinational study of multimorbid medical inpatients, we identified combinations of comorbidities associated with a prolonged LOS, quantified these associations, and assessed multiplicative effects of the different comorbidities on the LOS. We found the strongest association for diseases of white blood cells with hematological malignancy, whereas combinations including neurological diseases and chronic ulcer of skin increased the odds of a prolonged LOS by 137% to 265%. We also quantified the association of the LOS with the number of chronic diseases and the number of body systems involved and found a particularly strong association with the latter. Further studies would be warranted to assess a potential causal relationship between combinations of diseases and the LOS. Researchers could then first focus on patients with combinations of diseases associated with a prolonged LOS and that may be influenced to develop and test preventive interventions to shorten the LOS and thus reduce the burden of multimorbidity and hospitalizations.

## Author contributions

**Conceptualization:** Carole Elodie Aubert, Jacques Donzé.

**Formal analysis:** Niklaus Fankhauser, Andreas Limacher.

**Investigation:** Carole Elodie Aubert.

**Methodology:** Carole Elodie Aubert, Jacques Donzé.

**Supervision:** Jacques Donzé.

**Writing – original draft:** Carole Elodie Aubert.

**Writing – review & editing:** Jeffrey L Schnipper, Niklaus Fankhauser, Pedro Marques-Vidal, Jérôme Stirnemann, Andrew A Auerbach, Eyal Zimlichman, Sunil Kripalani, Eduard Vasilevskis, Edmondo Robinson, Joshua Metlay, Grant S Fletcher, Andreas Limacher, Jacques Donzé.

## Supplementary Material

Supplemental Digital Content
