# Supplementary material for: Association of patterns of multimorbidity with length of stay: A multinational observational study
Source: Medicine (Baltimore). 2020 Aug 21;99(34):e21650. doi: 10.1097/MD.0000000000021650 (PMC7447409; doi:10.1097/MD.0000000000021650)

**Association of patterns of multimorbidity with length of stay:**

a multinational observational study

**APPENDIX**

**Participating hospitals**

The following hospitals participated in the study:

1) University of California, San Francisco Medical Center, San Francisco, California, USA;

2) Harborview Hospital Medicine, University of Washington, Seattle, Washington, USA;

3) Vanderbilt University Medical Center, Nashville, Tennessee, USA;

4) Christiana Care Health System, Wilmington, Delaware, USA;

5) Hospital of the University of Pennsylvania, Philadelphia, Pennsylvania, USA;

6) Northwestern Memorial Hospital, Chicago, Illinois, USA;

7) Brigham and Women’s Hospital, Boston, Massachusetts, USA;

8) Department of General Internal Medicine, Inselspital, Bern University Hospital, Bern, Switzerland;

9) Department of Internal Medicine, CHUV, Lausanne University Hospital, Lausanne, Switzerland;

10) Department of Internal Medicine, HUG, Geneva University Hospital, Geneva, Switzerland;

11) Sheba Medical Center, Tel Hashomer, Israel.

**Eighteen body system categories of the Chronic Condition Indicator (CCI)**

The CCI classifies the chronic diseases into following eighteen body system categories:

1. infectious and parasitic diseases;
2. neoplasms;
3. endocrine, nutritional, and metabolic diseases and immunity disorders;
4. diseases of blood and blood-forming organs;
5. mental disorders;
6. diseases of the nervous system and sense organs;
7. diseases of the circulatory system;
8. diseases of the respiratory system;
9. diseases of the digestive system;
10. diseases of the genitourinary system;
11. complications of pregnancy, childbirth, and the puerperium;
12. diseases of the skin and subcutaneous tissue;
13. diseases of the musculoskeletal system;
14. congenital anomalies;
15. certain conditions originating in the perinatal period;
16. symptoms, signs, and ill-defined conditions;
17. injury and poisoning;
18. factors influencing health status and contact with health services.

**Categorization of diseases**

For clinical relevance, we further merged some categories of the Clinical Classification Software (CCS), creating following broader categories:

1. chronic heart disease (CHD): CCS 105-107 (cardiac dysrhythmias), CCS 100-101 (coronary heart disease), CCS 10 (nonhypertensive congestive heart failure), CCS 96 (heart valve disorder);
2. cerebrovascular diseases: CCS 109 and 111-112;
3. solid malignancies: CCS 11-36 and 41-42;
4. hematological malignancies: CCS 37-40;
5. arthropathy and arthritis: CCS 54 (gout and other crystal arthropathies), CCS 201 (infective arthritis and osteomyelitis), CCS 202 (rheumatoid arthritis and related disease), CCS 203 (osteoarthritis), CCS 204 (other non-traumatic joint disorders), CCS 205 (spondylosis, intervertebral disc disorders and other back problems);
6. osteoporosis and pathological fractures: CCS 206-207;
7. liver disease: CCS 6 and 150-151;
8. psychosis and schizophrenic disorders: CCS 70-71 and 659;
9. other nutritional, endocrine or metabolic disorder: CCS 51 and 58;
10. substance-related disorders: CCS 660-661.

**Detailed Figure 2**. Association between prolonged length of stay and a) number of chronic diseases and b) number of body systems involved.

**Abbreviations**: #, number of chronic diseases (a) / body systems (b); CI, confidence interval; OR, odds ratio.

**Legend**: Odds ratio (box) with 95% CI (lines) for prolonged length of stay, defined as a LOS longer than or equal to country-specific upper (75%) quartile.

251658240
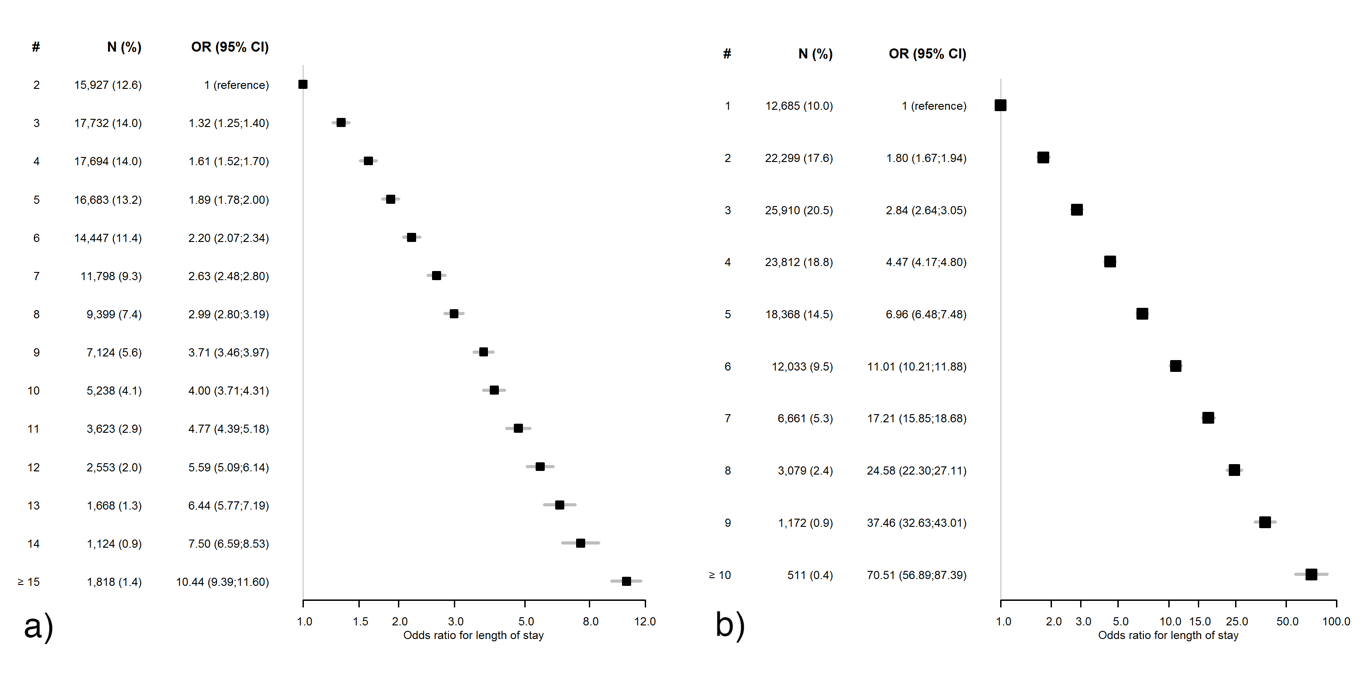

Supplement: Supplemental Digital Content [file medi-99-e21650-s001.docx]
